# Is space flight arrhythmogenic?

**DOI:** 10.3389/fphys.2023.1162355

**Published:** 2023-05-12

**Authors:** O. V. Popova, V. B. Rusanov

**Affiliations:** Institute of Biomedical Problems of the Russian Academy of Sciences, Moscow, Russia

**Keywords:** cardiovascular system (CVS), arrhythmia, space flight, cardiovascular event, electrocardiogram

How probable are arrhythmias in spaceflight? The International Space Station (ISS) has been in Low-Earth orbit (LEO) for more than 20 years. During this time, there have been no reports of pronounced arrhythmias in space crew members. Does this mean that there were no arrhythmias among the ISS crew members who were in LEO? Or were they not reported? On the one hand, the level of medical control in the selection and further screening of crews for space missions is high in all space agencies. On the other hand, routine activities on the ISS require significant skills and experience, and this leads to an increase in the age of space crew members, both males, and females. And this is a serious arrhythmogenic factor (Tank, 2005; Wittnich et al., 2013; Platts et al., 2014; Koenig and Thayer, 2016; Yoo and Fu, 2020). Not to mention that, undoubtedly, human cardiovascular physiology is not adapted to exist outside gravity (Shen and Frishman, 2019) and outside the magnetic field of Earth with increased levels of heavy ionizing radiation (Afshinnekoo et al., 2020).

It is known that since the late 1950s, 17 cases of atrial arrhythmias have been identified among 317 active and retired astronauts. Overall, the prevalence of arrhythmias, the most common of which was atrial fibrillation, was 5%, which is comparable to the prevalence of arrhythmias in the general population, but the age of astronauts is much younger (41–45 years). All of the above increases the likelihood of arrhythmias during long-term space flight (SF), as medical care will be limited (Khine et al., 2018). In addition, in this study, 48-h high-resolution Holter monitoring data were obtained before, during the flight and on the day of landing, and magnetic resonance imaging data before and after the 6-month SF. According to the results of this study, the volume of the left atrium temporarily increased after 6 months of SF (12 ± 18 mL; *p* = 0.03), while the function of the atria did not change, also 1 astronaut had a significant increase in supraventricular ectopic contractions, but none of them developed atrial fibrillation. Correspondingly, 6 months of stay in SF can cause temporary changes in the structure of the left atrium, which increase the risk of atrial fibrillation, but no episodes of atrial fibrillation were detected.

A Delp et al. (2016) study reported a 4–5 times higher risk of cardio-vascular disease (CVD) in Apollo astronauts compared to astronauts who never traveled beyond LEO. Therefore, due to the increase in the duration and range of planned SFs, the probability of cardiovascular system maladaptation and dysfunction increases as the influence of SF factors intensifies.

In the absence of Earth’s gravity there is a shift of fluid in cranial direction (about 2 L of fluid moves out of the legs) due to the decrease of intrathoracic pressure. Central venous pressure decreases within 1 day of being in microgravity, which is associated with a decrease in compression on the veins by muscles and internal organs (Hughson et al., 2018). Left ventricular end-diastolic volume increases concurrently with a decrease in central venous pressure (Buckey et al., 1996). Arterial pressure in microgravity is uniform throughout the body and thereby reduces cardiac strain and the physiological need for arterial pressure regulation mechanisms.

**FIGURE 1:**
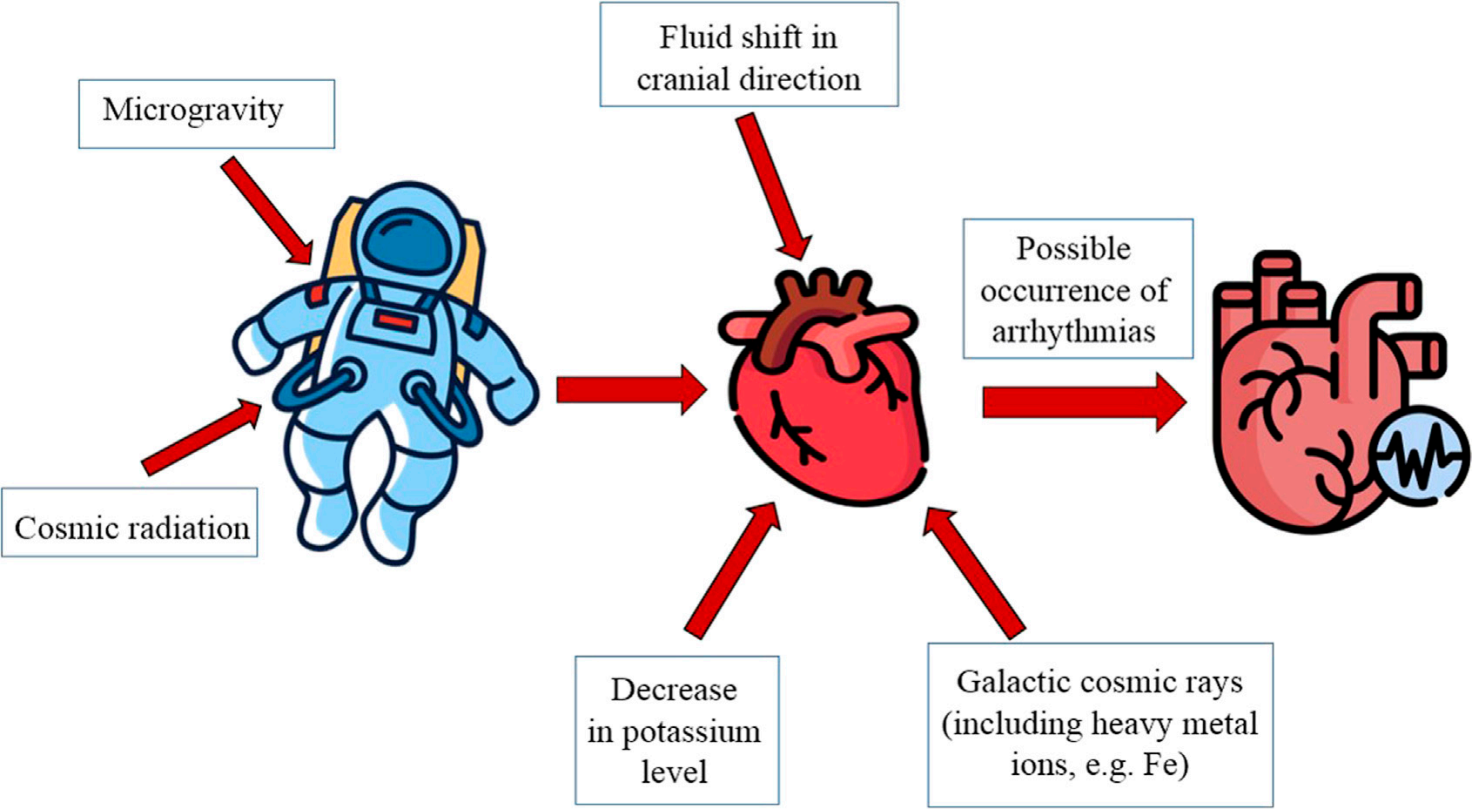
Factors of possible occurrence of arrhythmias in space flight.

Baroreceptor stimulation as a consequence of cardiac remodeling and increased cerebrovascular pressure due to redistributed fluid may affect neural and endocrine regulatory loops. This may lead to inhibition of the renin-angiotensin-aldosterone system and increased release of atrial natriuretic peptide (Frings-Meuthen P. et al., 2020). There is also a 10%–15% decrease in blood plasma volume (Leach C.S. et al., 1985), which has not been suggested to be due to increased diuresis but rather to a reduction in intrathecal pressure and an increase in vascular pressure in the upper body, which together promote transcapillary fluid movement into the upper body interstitial (Watenpaugh D.E., 2001). These responses stabilize during the first 2 weeks in space and persist thereafter in both short- and long-duration spaceflight (Leach C.S. et al., 1985; Garrett-Bakelman F.E. et al., 2019) (Figure 1).

Hughson R.L. et al. (2016) have shown increased aldosterone, which is a known cardiac remodeling factor, in 6-month spaceflight, with these hormones increasing more in female astronauts than in male astronauts. There is evidence from animal models and spaceflight that the levels of endogenous glucocorticoids are elevated (Yang J. et al., 2020). Similarly, in a study by Kumei Y. et al. (1985) it was found that glucocorticoid receptor mRNA levels were increased three to eight-fold on the 4th and 5th days of SF. However, these data were obtained in a cell-based study of osteoblasts.

Perhonen et al. (2001) showed that both after 10 days of SF and after 6 weeks of bed rest (BR), cardiac atrophy occurs as magnetic resonance imaging showed a 12% reduction in left ventricular mass. This study demonstrates that cardiac remodeling occurs under both SF and microgravity simulation conditions.

In addition, as early as 1985 (Bungo et al., 1987) a slight posterior wall reduction was observed together with a 28% decrease in stroke volume after SF of 5–8 days.

Left atrial volume was shown to increase transiently after 6 months of SF, but there was no difference in P-wave duration over time. And RMS20 decreased compared with the preflight period on all days except the day of landing (Khine et al., 2018).

Thus, an episode of ventricular tachycardia with a maximum frequency of 215 beats per minute and a duration of 14 min was recorded during the era of the MIR station. It has been suggested that the cause of tachycardia could be autonomic changes associated with changes in ventricular volume or mass of the heart (Fritsch-Yelle et al., 1998). Another medical problem during the MIR program was arrhythmias (Gontcharov et al., 2005).

In the investigation of Turchaninova et al. (2002) it was shown, that the most characteristic feature of electrocardiogram (ECG) in flight was the instability of ventricular repolarization elements. Their statistical analysis testified to significant decrease of T-beats amplitude, which started from the 1st month of the flight and was mainly diffuse with predominant changes in the leads, reflecting potentials of left ventricle posterolateral portions. The variability of the terminal part of the ventricular ECG complex was manifested by significant variations both in the magnitude, shape and direction of T-beats, and the number of leads in which these changes were detected, and the range of changes was rather wide: the presence of biphasic, biphasic and inverted T-beats in all leads of electrocardiogram.

During the 14-day BR it was noted that the alternation of microvolt T waves increased. So before BR micro alternations of T-waves were observed in 17% of the subjects (24 healthy men in total participated in the experiment), and after stay in analogue conditions of microgravity in 42. Also at the end of the 14-day stay in BR there was a tendency to increase potassium excretion (*p* = 0.06) compared with baseline values (Grenon et al., 2005).

Intensive regular physical activity can lead to morphological and electrical adaptations of the heart, commonly referred to as the “athlete’s heart”. There is a general consensus that athletes are more prone to sudden cardiac death (SCD) and arrhythmias than non-athletes (Link M.S. et al., 2010; Bisbal F. et al., 2012). Thus studies Basso, C. et al. (2020) have shown that SCD during sport activity account for a small but significant proportion, 5% of all SCDs. Long-term intensive endurance exercise is now considered to be an etiological factor in the development of supraventricular heart disorders, including atrial fibrillation, bradyarrhythmia and also atrioventricular block (D'Souza A. et al., 2019).

The main type of training on the Russian segment of the ISS for cosmonauts is a four-day cycle of locomotor training (Kozlovskaya I.B. et al., 2013), while the American segment for astronauts is characterized by resistance training (Loehr, J. A. et al., 2015), which is also used in the Russian segment. Thus, the avoidance of arrhythmias in SF requires individual and comprehensive training selection with a large variability of load parameters when performing resistance training.

Thought the Apollo 15 flight there were physiological abnormalities manifested by ectopic ECG activity (bigeminal premature ventricular contractions and premature atrial contractions) and unusual changes in exercise tolerance, which were thought to be related to decreased potassium content in the body and lack of potassium intake during the flight (Rossum et al., 1997). Plausibly, elevated urinary potassium levels may indicate inadequate potassium intake and may also indicate muscle atrophy. In addition, it is known that during diastole, potassium currents mainly maintain the resting potential (RP) of cardiomyocyte membranes; accordingly, changes in potassium concentration (especially extracellular) can directly alter cardiomyocyte RP in SF (Jeevaratnam et al., 2018; King et al., 2021). Therefore, potassium intake must be strictly monitored for future long-duration SF.

Sleep disturbance is a serious problem for space travelers. It is known that under spaceflight conditions there is a shortening of sleep time, moreover, the number of awakenings increases, and the amount of slow-wave sleep and REM sleep decreases (Dijk D.J. et al., 2001). Extreme space conditions, heavy workload may seriously disturb not only sleep but also circadian rhythms. And mismatch between circadian clock and sleep also affects the cardiovascular system, thus increasing the risk of cardiovascular disease (Guo, JH. et al., 2014).

Prolonged time in outer space will inevitably increase the health risks to space crew due to exposure to galactic cosmic rays and solar particles. Although the dose of cosmic radiation is lower than in radiation therapy patients, epidemiological evidence suggests an increased risk of late CVD even at low radiation doses (Hughson et al., 2018).

Soucy et al. (2011) examined vascular and endothelial function in healthy rats exposed to a single whole-body dose of 56 Fe (0, 0.5, or 1 Gy). *In vivo* aortic stiffness and *ex vivo* aortic tensile response, as indicators of chronic vascular damage, were measured 6 and 8 months after irradiation. Rats irradiated with 1 Gy 56Fe showed a significant increase in aortic stiffness as measured by pulse wave velocity. Aortic rings of irradiated rats showed impaired endothelium-dependent relaxation, which is consistent with endothelial dysfunction. According to the results of the research (Yan et al., 2014; Ramadan et al., 2016; Seawright et al., 2019) we can conclude that exposure to 56 Fe ions affects myocardial remodeling, increase of left ventricle end-diastolic volume, and fibrosis in mice.

In conclusion, we suggest that SF may be arrhythmogenic, as being outside LEO can be accompanied by exposure to various particles contained in cosmic radiation, which can lead to the development of various CVD such as accelerated atherosclerosis, microvascular damage, and myocardial fibrotic remodeling (Meerman et al., 2021), in addition, the duration of flights (at the moment, the flights mostly last 6 months, and the flight to Mars is supposed to last more than 2 years, taking into account the stay and flight in both directions) and the age of astronauts are increasing, respectively increasing the risk of late CVD and the likelihood of atrial fibrillation because increase of left ventricle end-diastolic volume occurs due to fluid redistribution (Chow et al., 2012; Chen et al., 2017). Despite the data of registration of arrhythmias in SF, arrhythmias can have not only a pathological character, but also a neurogenic nature. Unfortunately, there is no data on the etiology of arrhythmias in SF at the moment. So, controls of arrhythmia cases are mandatory.
